# Retrospective evaluation of the effectiveness of indoor residual spray with pirimiphos‐methyl (Actellic) on malaria transmission in Zambia

**DOI:** 10.1186/s12936-021-03710-5

**Published:** 2021-04-01

**Authors:** Joseph Keating, Joshua O. Yukich, John M. Miller, Sara Scates, Busiku Hamainza, Thomas P. Eisele, Adam Bennett

**Affiliations:** 1 Center for Applied Malaria Research and Evaluation, Department of Tropical Medicine, School of Public Health and Tropical Medicine, Tulane University, 1440 Canal Street, Suite 2320, New Orleans, 70112 USA; 2 PATH Malaria Control and Elimination Partnership in Africa (MACEPA), National Malaria Elimination Centre, Chainama Hospital College Grounds, Lusaka, Zambia; 3 National Malaria Elimination Centre, Ministry of Health, Lusaka, Zambia; 4 Global Health Sciences, University of California, San Francisco, CA USA

**Keywords:** Pirimiphos-Methyl (Actellic), Malaria, Zambia, Indoor Residual Spray, mSpray

## Abstract

**Background:**

Widespread insecticide resistance to pyrethroids could thwart progress towards elimination. Recently, the World Health Organization has encouraged the use of non-pyrethroid insecticides to reduce the spread of insecticide resistance. An electronic tool for implementing and tracking coverage of IRS campaigns has recently been tested (mSpray), using satellite imagery to improve the accuracy and efficiency of the enumeration process. The purpose of this paper is to retrospectively analyse cross-sectional observational data to provide evidence of the epidemiological effectiveness of having introduced Actellic 300CS and the mSpray platform into IRS programmes across Zambia.

**Methods:**

Health facility catchment areas in 40 high burden districts in 5 selected provinces were initially targeted for spraying. The mSpray platform was used in 7 districts in Luapula Province. An observational study design was used to assess the relationship between IRS exposure and confirmed malaria case incidence. A random effects Poisson model was used to quantify the effect of IRS (with and without use of the mSpray platform) on confirmed malaria case incidence over the period 2013–2017; analysis was restricted to the 4 provinces where IRS was conducted in each year 2014–2016.

**Results:**

IRS was conducted in 283 health facility catchment areas from 2014 to 2016; 198 health facilities from the same provinces, that received no IRS during this period, served as a comparison. IRS appears to be associated with reduced confirmed malaria incidence; the incidence rate ratio (IRR) was lower in areas with IRS but without mSpray, compared to areas with no IRS (IRR = 0.91, 95% CI 0.84–0.98). Receiving IRS with mSpray significantly lowered confirmed case incidence (IRR = 0.75, 95% CI 0.66–0.86) compared to no IRS. IRS with mSpray resulted in lower incidence compared to IRS without mSpray (IRR = 0.83, 95% CI 0.72–0.95).

**Conclusions:**

IRS using Actellic-CS appears to substantially reduce malaria incidence in Zambia. The use of the mSpray tool appears to improve the effectiveness of the IRS programme, possibly through improved population level coverage. The results of this study lend credence to the anecdotal evidence of the effectiveness of 3GIRS using Actellic, and the importance of exploring new platforms for improving effective population coverage of areas targeted for spraying.

## Background

Vector control through the use of long-lasting insecticidal nets (LLINs), and the use of indoor residual spraying (IRS), have been a cornerstone to recent malaria control success [1, 2].

However, widespread insecticide resistance to pyrethroids is a major threat to continued progress towards elimination [3]. Recently, the Global Plan for Insecticide Resistance Management (GPIRM) of the World Health Organization (WHO) has encouraged the use of non-pyrethroid insecticides as a part of GPIRM best practices [4] to reduce the spread of insecticide resistance.

Programs considering new or additional insecticides for IRS lack rigorous field-based evidence on the effectiveness of next (3rd) generation pyrethroid-free insecticides for IRS (3GIRS). In addition, information on the combination of vector control tools is needed, given the high cost of program rollout. As the effectiveness of pyrethroid-based insecticides is well documented for both LLINs and IRS [1, 2, 5], and IRS is considered a cost-effective vector control intervention in many settings [6], a logical next step would be to assess whether adding new 3GIRS chemicals improves malaria prevention and control practices.

The WHO recommends a threshold of 85% community-level coverage of IRS to maintain effectiveness. A study conducted on Bioko Island demonstrated that if the community is covered at or above an 80% threshold, houses not receiving IRS see the same benefits as their sprayed neighbors through a community effect [7]. Therefore, effective spatial coverage of IRS is critical; the location of all structures in the program area must be enumerated or mapped to increase the likelihood that the 85% threshold is reached. Until recently, this was done by teams of enumerators, a time consuming and costly process that often led to a number of structures being missed. While this is better than not enumerating, this can result in high ‘reported operational coverage’ (i.e. the reported number of structures sprayed out of the total structures enumerated), but poor ‘effective population coverage’ (i.e. the number of structures sprayed out of the total eligible structures actually present within an area).

A new electronic tool for implementing and tracking coverage of IRS campaigns in Zambia has recently been tested. This tool, mSpray (now called REVEAL), is a mobile and desktop-based platform that utilizes satellite imagery to improve the accuracy and efficiency of the enumeration process by ensuring all eligible structures are identified [8]. While mSpray technology should, theoretically, increase ‘effective population coverage’ and thus the effectiveness of IRS due to the improved community-effect of the intervention, it may yield lower ‘reported operational coverage’ due to the identification of additional structures that increase the target-area denominator. By recording and tracking spray operation data at the structure level, a more thorough accounting of both sprayable and non-sprayable structures is achieved.

The Zambian Government has identified malaria control as a priority, with the vision of a malaria elimination [9]. Since the reintroduction of IRS in Zambia in 2000, IRS has been a cornerstone of malaria vector control in the country, effectively scaling up from 5 districts in 2003 to all districts (72 in 2011, now 117 as of 2018) by 2011 [10]. From 2000 to 2009, vector control relied exclusively on DDT and pyrethroids, both of which share the same mode of action. In 2009, resistance to both insecticide classes was detected, prompting the formation of the Insecticide Resistance Management Technical Working Group (IRMTWG) in 2010 [11, 12]. In 2011, the use of DDT was discontinued, and carbamates and organophosphates were introduced. Due to growing concerns of documented resistance in all areas of Zambia to all classes of insecticides, except the organophosphates, Actellic 300CS, a microencapsulated formulation of the organophosphate insecticide pirimiphos-methyl, was introduced in 2013 [10]. While Actellic 300CS is thought to be more effective than pyrethroid-based chemicals because of insecticide susceptibility, it is considerably more expensive [13–15]. A study in Migori County, Kenya recently demonstrated that a single application of IRS with Actellic 300CS provided ten months protection, resulted in the near elimination of the primary malaria vector *Anopheles funestus*, and reduced malaria case counts among febrile outpatients [12]. However, empirical evidence of the effectiveness of Actellic 300CS on human health outcomes is quite limited and programmes and donors are increasingly looking for rigorous evidence of effectiveness when considering new interventions [16].

The use of LLINs, IRS and investigational vector control tools such as 3GIRS with Actellic 300CS in Zambia provides an opportunity for a retrospective evaluation of the effectiveness of 3GIRS across different scenarios. The purpose of this paper is to retrospectively analyse cross-sectional observational data to provide evidence of the epidemiological effectiveness of having introduced Actellic 300CS into IRS programmes across Zambia as well as the effectiveness of the mSpray platform for increasing spray coverage.

## Methods

### Study setting

This study was conducted in Luapula, Northern, Muchinga, and Eastern provinces in Zambia. Malaria transmission in these provinces peaks from December to June following the seasonal rains. The Zambia National Malaria Elimination Centre (NMEC) has successfully scaled up the main WHO-recommended malaria control interventions (i.e. LLINs, IRS, and improved access to prompt effective treatment of malaria) over the past decade and is now considering alternative strategies to further reduce the malaria burden [17]. Table 1 lists study vector control coverage and parasite prevalence by study province [18].

**Table 1:** Study setting LLIN, IRS and under 5-year-old malaria prevalence status in 2015

*Province*	Luapula	Northern	Muchinga	Eastern
%HH LLIN ownership	88.6	78.9	82.2	94.5
% HH sprayed with IRS	31.6	17.5	27.7	56.0
Malaria prevalence (< 5)	32.5	27.6	21.4	12.7

Mass ITN campaigns are conducted periodically throughout the country, with campaigns occurring in 2014 and late 2017. IRS campaigns are conducted annually, ideally from October to November, just prior to the onset of the seasonal rains. The 2018 MIS reported 80% of Zambian households owning at least one ITN, up from 62% to 2008; and 34.8% of Zambian households having been sprayed with insecticide in the previous 12 months up from 14% to 2008 [19].

### PMI Africa indoor residual spraying project (AIRS) and mSpray implementation

In 2014, AIRS Zambia identified 40 high burden districts in 5 selected provinces, and subdistrict catchment areas were targeted for spraying with pirimiphos-methyl [20]. Criteria for inclusion included areas where recent national prevalence surveys suggest high burden, operational areas where PMI operates and programmatic information on insecticide resistance patterns [21]. The mSpray platform was used in 7 districts in Luapula Province. The mSpray platform is a tool used to improve IRS enumeration, targeting, and spraying. It is a cloud-based data recording and management system that allows spray personnel to collect spray data and GPS coordinates electronically in the field using a tablet. Data are submitted to the cloud for immediate viewing of spray campaign progress in relation to satellite-based and field-verified structure enumeration data. Spraying began in 2014 and was repeated in 2015 and again in 2016. Spraying was only conducted in Central Province in 2014 and 2015, and in a smaller number of catchment areas, and was excluded from the analysis.

### Study design

An observational study design was used to assess the relationship between IRS exposure and confirmed malaria case incidence. The primary outcome was confirmed outpatient (OPD) malaria case incidence among all ages during the high transmission season (Dec–June) between 2013 and 2017: defined as the number of OPD confirmed malaria cases at the health facility catchment level, standardized per catchment population, as reported to the health management information system (HMIS). Case confirmations included both malaria RDT and microscopy reporting. Exposure was defined at the health facility catchment level by having IRS activities conducted in the preceding season.

### Data sources

Data on IRS coverage were collected from the AIRS and mSpray programmes: defined as the total number of households sprayed by date within target areas. Monthly enhanced vegetation indices (EVI) from MODIS satellite imagery (https://lpdaac.usgs.gov/products/mod13a3v006/) and monthly rainfall data from the CHIRPs dataset (https://www.nature.com/articles/sdata201566) were also extracted at the health facility level for those facilities with available geographical coordinates as control variables in the analysis.

### Data analysis

All health facility catchment areas within the study area were categorized as either having received IRS using the mSpray platform, receiving IRS without mSpray, or receiving no IRS, for the spray seasons 2014, 2015, and 2016. For descriptive comparisons across all catchment areas, raw operational coverage estimates were calculated by dividing the numbers of structures reported sprayed by the programme, by the numbers of structures reported as found. As the numbers of structures found were sometimes calculated differently for mSpray and non-mSpray areas, the total number of structures sprayed per population for each health facility catchment was also compared; this value was calculated as the total number of reported structures sprayed divided by the catchment area population as estimated from administrative records.

Catchment area health facilities were matched to the number of confirmed malaria cases per month over 2013–2017, the number of tests conducted per month over 2013–2017, and the estimated catchment population size. The total number of confirmed malaria cases was calculated over the high transmission season (December–June, following each IRS spray campaign) for each facility. Facilities that had poor reporting (data for fewer than 3 of the 7 high transmission months available) were removed from the analysis. December–June incidence rates per 1000 were calculated by dividing the total December–June confirmed cases by the catchment population and multiplying by 1000. Total rainfall data were extracted for facilities with geographical coordinates and lagged by two months (November–April), and the mean enhanced vegetation index (EVI) was calculated for each facility and lagged by one month (December–May).

A random effects Poisson model was used to quantify the effect of IRS (with and without use of the mSpray platform) on January–June confirmed case incidence at the catchment level over the period 2013–2017. The primary effect was estimated using a categorical dummy variable representing “no IRS”, “IRS”, or “IRS with mSpray” for each health catchment area. Province and year fixed effects were included to control for regional and temporal trends. The total number of tests conducted controlled for variable testing rates, and the previous year’s total confirmed cases were included to control for temporal autocorrelation. Rainfall and EVI were included as covariates to control for the overall propensity of the area to harbour mosquito populations.

## Results

**Fig. 1:**
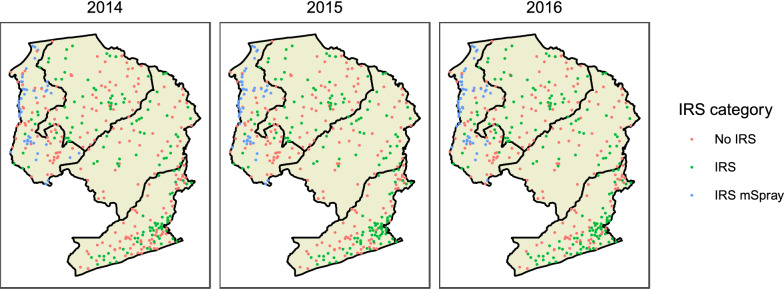
The distribution of health facility intervention assignment by year in the four provinces of Zambia included in the analysis. The assignment to IRS category was based on IRS planning meetings with district health offices and PMI

IRS was conducted in a total of 283 catchment areas from 2014 to 2016 and expanded over time: 219 in 2014, 243 in 2015, and 255 in 2016. mSpray was used at 66 health facility catchment areas in Luapula province from 2014 to 2016: 51 received mSpray in 2014, 52 in 2015, and 53 in 2016. After linking with geographical and environmental data, a total of 185 IRS catchment areas (48 mSpray) were available for the regression analysis in 2014; 203 (50 mSpray) in 2015; and 212 (48 mSpray) in 2016 (Table 2). A total of 157 health facilities that received no IRS during this period were available for comparison in the regression analysis (Fig. 1).

**Table 2:** Number of health facilities included in the analysis by year and IRS status

Category	2013	2014	2015	2016	2017
*Included in descriptive analysis*					
No IRS	481	481	262	238	226
IRS	0	0	168	191	202
IRS w/ mSpray	0	0	51	52	53
*Included in regression*					
No IRS	392	392	207	189	180
IRS	0	0	137	153	164
IRS w/ mSpray	0	0	48	50	48

**Fig. 2:**
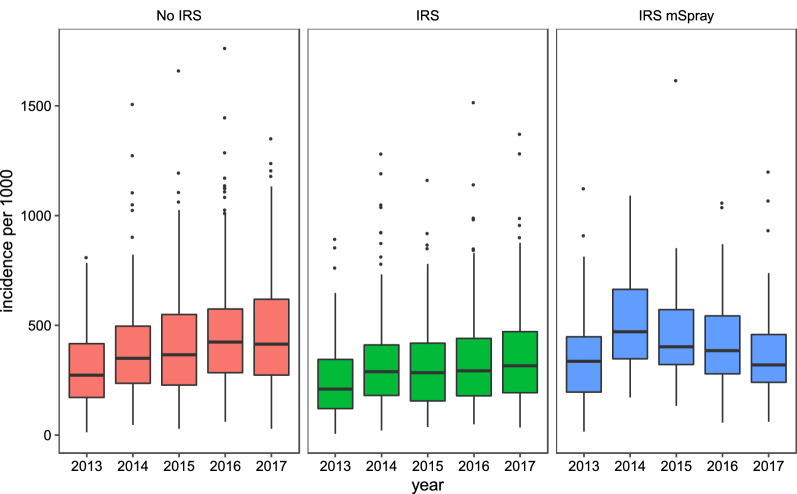
Confirmed malaria case incidence per 1000 (Dec–Jun) by year for facilities with **a** no IRS, **b** IRS, and **c** IRS with mSpray

Confirmed malaria case incidence per 1000 rose slightly over the period of observation 2013–2017. Confirmed case incidence by year leveled out in those receiving IRS and decreased in 2016 and 2017 in those receiving IRS with mSpray (Fig. 2). Reported operational coverage estimates were high for both IRS and IRS/mSpray, but slightly higher for IRS without mSpray (Table 3). Conversely, the number of structures sprayed per population was slightly higher for IRS with mSpray across all years (Table 3).

**Table 3:** Coverage estimates and confidence intervals by IRS type

Coverage estimate	2014	2015	2016
*Operational coverage*			
IRS	94.7 (93.6–95.9)	94.3 (93.2–95.4)	92.4 (91.4–93.4)
IRS with mSpray	87.0 (83.5–90.5)	95.0 (93.8–96.3)	88.8 (87.4–90.1)
*Structures per population*			
IRS	0.13 (0.11–0.14)	0.17 (0.15–0.18)	0.17 (0.15–0.20)
IRS with mSpray	0.14 (0.11–0.17)	0.19 (0.14–0.23)	0.21 (0.16–0.25)

Receiving IRS appears to be associated with reduced confirmed malaria incidence; the incidence rate ratio (IRR) was lower in areas with IRS but without mSpray, compared to areas with no IRS, but the effect size was smaller (IRR = 0.91, 95% CI 0.84–0.98) (Table 4). Receiving IRS with mSpray was significantly protective against confirmed case incidence (IRR = 0.75, 95% CI 0.66–0.86) compared to no IRS. Compared directly, IRS with mSpray resulted in lower incidence than IRS without mSpray (IRR = 0.83, 95% CI 0.72–0.95).

**Table 4:** Random effects Poisson regression results

Parameter	Incidence rate ratio (IRR)	95% Confidence Interval	P-value
*Province*			
Eastern (ref.)			
Luapula	1.72	1.49–1.98	< 0.001
Muchinga	1.27	1.11–1.45	0.001
Northern	1.04	0.89–1.23	0.621
*IRS*			
None (ref.)			
IRS	0.91	0.84–0.98	0.019
IRS w/ mSpray	0.75	0.66–0.86	< 0.001
Rainfall	1.00	1.00–1.00	0.017
EVI	1.50	0.88–2.56	0.136
*Year*			
2014 (ref.)			
2015	1.12	1.04–1.21	0.004
2016	1.16	1.06–1.24	0.001
2017	1.11	1.00–1.19	0.050
Total tested	1.00	0.99–1.00	0.260
Total confirmed previous year	0.99	0.99–1.00	0.551

## Discussion

This analysis used multiple datasets linked spatially and temporally to assess the relationship between IRS using Actellic-CS, IRS using the mSpray platform, no IRS, and malaria incidence at a subnational level in Zambia. Total confirmed malaria case incidence has increased slightly over this period as diagnostic capacity has also increased [19]. However, this analysis showed a consistent association between implementation of IRS and lower confirmed malaria case incidence, and a stronger association where mSpray was used; the use of 3 GIRS insecticides may be enhancing the observed effect, likely due to vector increased susceptibility to the insecticide. The mSpray electronic tool also appeared to improve the effectiveness of IRS. The greater effect observed in IRS areas using mSpray may be due to achieving more even, and overall higher, population level household coverage.

There are potential biases in operational coverage reporting when reliant on self-reported programme data. For example, spraying of houses requires a high level of training to produce a consistent and appropriate coating of insecticide on walls of varying sizes and materials. When operational definitions are used to define spray coverage it is clear that some houses or structures defined as sprayed will actually be inadequately sprayed with insecticide, perhaps due in part to a failure to spray all of the appropriate rooms or surfaces. In addition, without adequate supervision, non-sprayed structures may be reported as sprayed. Behavioural factors and demand may also contribute to bias in reporting coverage. Individuals may refuse entry to spray teams or be absent at the time of the visit; individuals may also re-paint or re-plaster walls shortly after allowing their homes to be sprayed [22]. Little quantitative information is available on these phenomena, but what is available indicates that large fractions of the population may engage in behaviour that prevents or reduces IRS effect [22].

Evaluation of IRS programmes from observational data has proven challenging because in most cases the programme is highly targeted to higher malaria burden areas, leading to large amounts of endogeneity in observational studies [23]. Though several studies have used alternative approaches to this [24], standard statistical methods are likely to result in counterintuitive estimates of positive associations between malaria prevalence or incidence and IRS application largely because of this kind of targeting, though targeting is highly desirable from a control perspective. Alternative study designs such as interrupted time series, differences-in-differences, and spatio-temporal modelling approaches can, sometimes, mitigate this kind of endogeneity and yield sensible estimates of IRS effects from observational data.

The effects of IRS with Actellic in this setting were much larger than the estimated effect of IRS with other chemicals, especially pyrethroids, observed elsewhere. This may be due to insecticide resistance reducing the susceptibility of the local vectors to IRS chemicals other than Actellic, the extended duration of effectiveness of Actellic on house walls compared to the other IRS chemicals used, or a combination of both these factors. This additional effect comes, however, at an increased cost. Actellic is currently a much higher priced active ingredient than all other WHO-approved IRS active ingredients. The extent to which this extra effectiveness would be considered beneficial relative to other vector control alternatives will require more detailed investigation of the cost and cost-effectiveness of IRS with Actellic as compared improved LLIN coverage, use of larvicides or improving access to prompt and effective care. In this context, this study has estimated an effectiveness of IRS with Actellic that is similar to that found for LLINs under conditions of limited or no insecticide resistance [1], although any variations in insecticide resistance from the previously used insecticide could be important.

While this study suggests a positive impact of 3GIRS on malaria incidence, the results should be interpreted with some caution, as the mSpray intervention was done in one province only, so there may be an underlying provincial effect in place. Secondly, this was an observational study; despite attempts to mitigate the effects of endogeneity due to IRS targeting, and to adjust for all appropriate confounding factors, the estimate of IRS effects found here are the result of associations rather than the results of a controlled experiment. Third, several unnamed ‘other’ facility catchment areas received IRS but could not be identified through the available data sources. Some of these facilities may have erroneously been included in the comparison group, leading to information bias resulting from misclassification. However, as this would tend to bias results of effect toward null results, these results are likely conservative. Fourth, an independent comparison of population level IRS coverage across areas using different reporting schemes to quantitatively assess whether coverage drove the greater effect observed in mSpray areas was lacking. Lastly, there was limited data on IRS in the period before the intervention activity, so it is not known if these catchment areas reflect true baseline values or not.

## Conclusions

IRS using Actellic-CS, the first approved and widely used 3GIRS chemical, appears to be associated with reduced malaria incidence in Zambia. The use of the mSpray tool appears to improve the effectiveness of the IRS programme, possibly through allowing for better targeting and improved population level coverage. The results of this study lend credence to the anecdotal evidence of the effectiveness of 3GIRS using Actellic, and the importance of exploring new platforms for improving effective population coverage of areas targeted for spraying.

## Data Availability

The datasets used and/or analysed during the current study are available from the corresponding author on reasonable request.

## Abbreviations

IRS: Indoor residual spraying
IRR: Incidence rate ratio
3GIRS: Third generation pyrethroid-free insecticides for IRS
LLINs: Long-lasting insecticidal nets
WHO: World Health Organization
GPIRM: Global Plan for Insecticide Resistance Management
NMEC: National Malaria Elimination Centre
ITN: Insecticide-treated bed net
PMI: President’s Malaria Initiative
AIRS: PMI Africa Indoor Residual Spraying Project
OPD: Outpatient department
HMIS: Health management information system
EVI: Enhanced vegetation index
